# Case report: Aromatic L-amino acid decarboxylase deficiency in three patient cases from the Kingdom of Saudi Arabia

**DOI:** 10.3389/fped.2022.1016239

**Published:** 2023-01-16

**Authors:** Musaad Abukhaled, Laila Alrakaf, Hesham Aldhalaan, Suad Al Yamani

**Affiliations:** Department of Neurosciences, King Faisal Specialist Hospital and Research Centre, Riyadh, Saudi Arabia

**Keywords:** AADC deficiency, Saudi Arabia, hypotonia, oculogyric crises, developmental delay, delayed diagnosis, seizures, case report

## Abstract

Aromatic L-amino acid decarboxylase (AADC) deficiency is an ultra-rare and often severe neurometabolic disorder resulting from variants in the dopa decarboxylase (*DDC*) gene. A timely diagnosis is critical to prevent secondary complications, promote development, and optimize outcomes from future innovative treatment options, such as gene therapy. This article describes three patients with AADC deficiency managed in the Kingdom of Saudi Arabia (KSA). All three patients had homozygous variants within the *DDC* gene, including one novel gene variant (c.245G > A, p.Arg82Glu), and presented with symptoms from birth. In all cases, a diagnostic delay was observed owing to non-specific signs and symptoms, a lack of disease awareness among primary care physicians, and delays associated with outsourcing of genetic tests. All three patients were managed by a multidisciplinary team at a specialist tertiary center. Clinical outcomes for all three cases were poor, with one patient passing away at 3 years of age and the other two patients continuing to experience substantial disability and poor quality of life. There is an urgent need to raise awareness and improve diagnostic testing for rare diseases such as AADC deficiency in the KSA in order to improve outcomes, particularly as innovative disease-targeting therapies become available.

## Introduction

Aromatic L-amino acid decarboxylase (AADC) deficiency is a rare, autosomal recessive, neurometabolic disorder resulting from pathological variants within the dopa decarboxylase (*DDC*) gene (1). It was first described in 1990; since then, at least 135 cases have been reported in the literature (2, 3). The global prevalence is unknown but a pilot newborn screening program in Taiwan indicated incidence rates of AADC deficiency to be 1:32,000 (4), whereas the incidence is estimated to range from 1 in 42,000–90,000 in the United States to 1 in 116,000 in Europe, and 1 in 162,000 in Japan (5–8).

The AADC enzyme is required for the final step in the synthesis of monoamine neurotransmitters, and deficiency results in a loss of dopamine, serotonin, epinephrine, and norepinephrine production, leading to severe physical and intellectual disabilities and risk of premature death (1, 9, 10). In most reported cases, patients have no or very limited attainment of developmental milestones (9). The symptoms and signs of AADC deficiency can be highly variable; however, the majority of patients present with hypotonia, global developmental delay and oculogyric crises. Other signs and symptoms may include autonomic dysfunction, sleep disturbances, feeding and swallowing difficulties and behavioral problems (3, 10–12). A syndromic intellectual disability phenotype which included developmental delay, intellectual disability and autonomic symptoms but lacked hypotonia or oculogyric crises has also been reported (13).

The three key diagnostic tests for AADC deficiency include: (i) genetic testing to identify pathogenic variants in the *DDC* gene; (ii) biochemical evaluations to measure the levels of neurotransmitter metabolites in cerebrospinal fluid (CSF) with a typical AADC deficiency profile including low CSF levels of 5-hydroxyindoleacetic acid, homovanillic acid, and 3-methoxy-4-hydroxyphenylglycol, normal CSF pterins, and high CSF levels of 3-O-methyldopa, L-DOPA, and 5-hydroxytryptophan; and (iii) assessment for decreased AADC enzyme activity in plasma. AADC deficiency is not associated with any specific brain magnetic resonance imaging (MRI) patterns; however, MRI is commonly used to rule out differential diagnoses with other conditions when a patient presents with neurodevelopmental delay. Similarly, electroencephalography (EEG) is not essential for diagnosis of AADC deficiency, although it can be useful to differentiate oculogyric crises from epileptic seizures if there is clinical suspicion of epilepsy (9). Because the clinical symptoms of AADC deficiency are non-specific, diagnosis is often delayed and patients are frequently misdiagnosed with other more common conditions, such as cerebral palsy or seizure disorders (14). Lack of awareness of AADC deficiency among primary care physicians, pediatricians, and pediatric neurologists can also contribute to diagnostic delays owing to low clinical suspicion for the disease (6).

Until recently, there was no cure for AADC deficiency, instead a multidisciplinary management approach was recommended to prevent secondary complications and to promote development. This should include physiotherapy, speech therapy, occupational therapy, and feeding and nutritional assessment, as well as psychological treatment and support. Dopamine agonists, monoamine oxidase inhibiters, and pyridoxine/pyridoxal phosphate are currently recommended as first-line medications for affected patients; anticholinergics, benzodiazepines, and melatonin can be used to control symptoms. For most patients, these medications appear to offer limited clinical benefits (9); however, AADC deficiency is an attractive target for gene therapy, and recently completed studies have demonstrated that delivery of a functional copy of the *DDC* gene to the basal ganglia to primarily target the dopaminergic systems using an adeno-associated viral vector leads to clinical improvements (10, 15–17). These trials found that improvements following gene therapy correlated with younger age, emphasizing the need for earlier diagnosis of AADC deficiency (15, 16). Newborn screening can potentially be used to expedite a diagnosis given that levels of 3-O-methyldopa (3-OMD) in dried blood spots are elevated in affected individuals (4).

**Figure 1:**
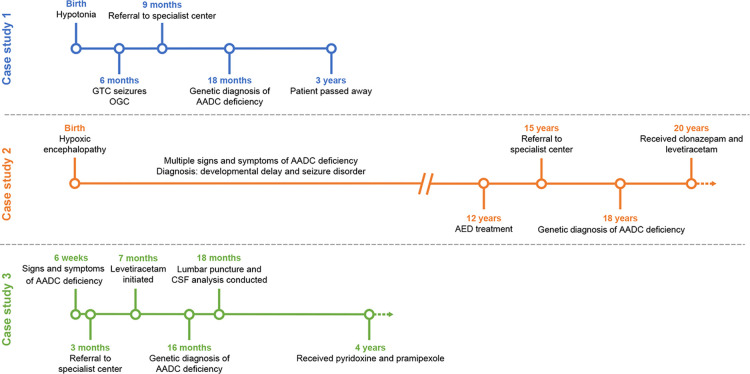
Timeline for the diagnosis and management of the three case study patients. AADC, aromatic L-amino acid decarboxylase; AED, anti-epileptic drug; CSF; cerebrospinal fluid; GTC, global tonic-clonic; OGC, oculogyric crises.

We present three patients who were diagnosed with AADC deficiency, one patient with a novel disease-causing variant, and managed at the King Faisal Specialist Hospital and Research Centre (KFSH-RC) in the Kingdom of Saudi Arabia (Figure 1). The objective of this case report is to raise awareness of AADC deficiency among physicians in a region where, owing to the prevalence of consanguineous marriages, case numbers may be underreported.

## Case presentations

### Patient case 1

**Figure 2:**
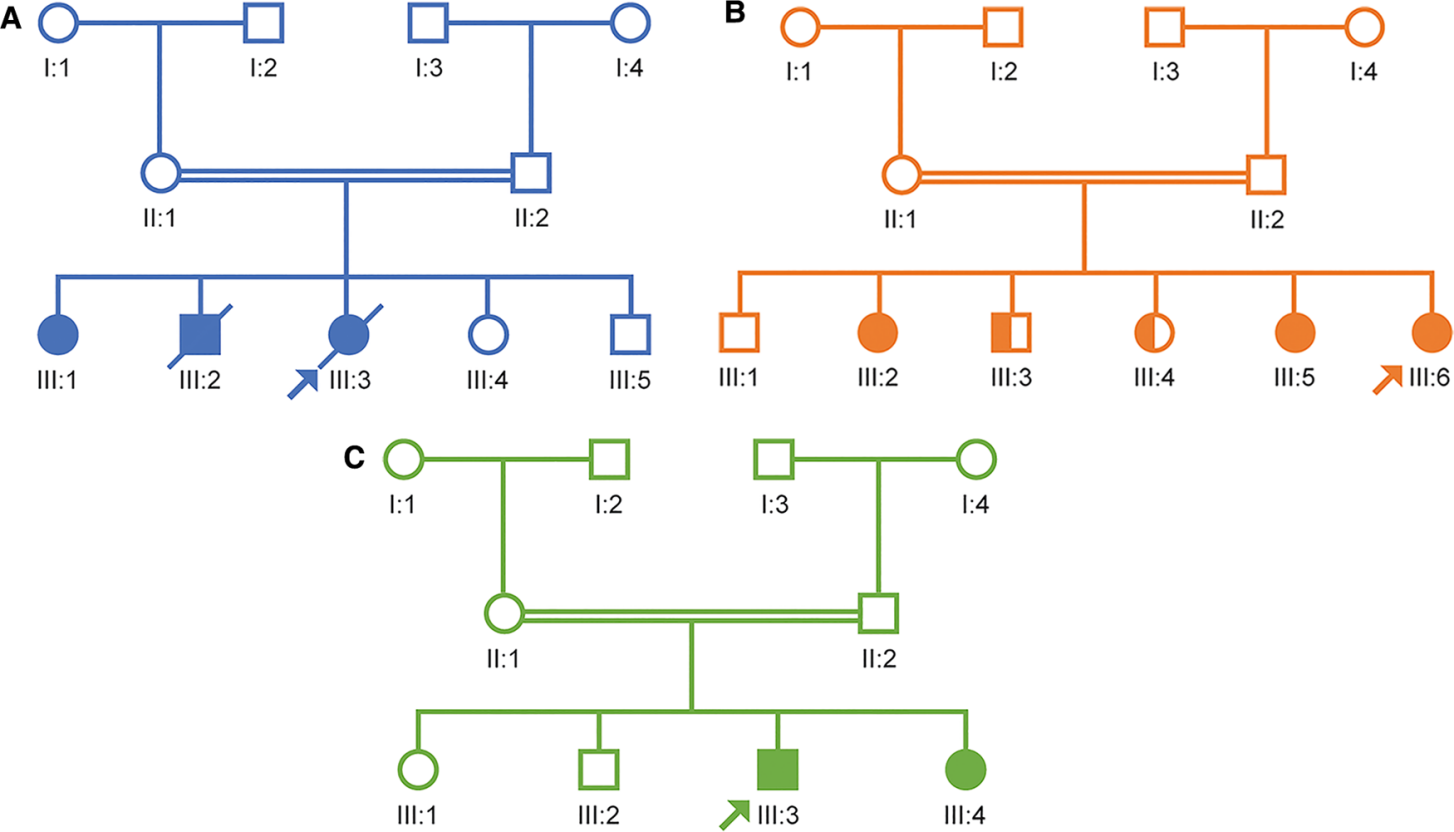
Pedigree diagrams for the three case study patients. (**A**) Case study 1. (**B**) Case study 2. (**C**) Case study 3.

This patient was a girl born to consanguineous parents (first-degree cousins) with a potential family history of AADC deficiency. The patient had one sibling diagnosed with cerebral palsy (now deceased) and a second sibling with a similar presentation who currently has a tracheostomy on conservative management. The second sibling has subsequently undergone genetic testing and been diagnosed with AADC deficiency. An additional sibling died *in utero* (Figure 2A).

This patient presented with hypotonia at birth but was discharged from the local hospital against medical advice. At 6 months of age, she began to experience generalized tonic-clonic seizures with oculogyric crises that lasted for a few minutes and occurred 2–3 times per week. The patient also exhibited a range of additional signs and symptoms, including global developmental delay, jerking of limbs, which are now believed to be dystonic attacks, excessive crying, insomnia, severe oropharyngeal dysphagia, and silent aspiration on thin liquid as well as autonomic symptoms including drooling, excessive sweating and hyperthermia.

The patient was initially treated with supportive management and anti-epileptic medications. At 9 months of age, she was referred for further evaluation at KFSH-RC and underwent a brain MRI, an EEG, and a lumbar puncture. At this time, genetic testing was requested, and the sample was sent for whole exome sequencing and to an epilepsy gene panel (Mayo Clinic Laboratories). Findings from the MRI scan were unremarkable, although intermittent slow activity was reported on the EEG. CSF analysis revealed a neurotransmitter profile consistent with AADC deficiency, including low levels of 5-hydroxyindoleacetic acid (5-HIAA; 6 nmol/l) and homovanillic acid (HVA; 38 nmol/L), and elevated 3-OMD (>2,500 nmol/L). Pyridoxal phosphate levels were normal (41 nmol/L), indicating that the patient did not have pyridoxamine-5-phosphate deficiency. Genetic testing results were received when the patient was 18 months old and revealed a previously unpublished homozygous variant in exon 3 of the *DDC* gene (c.245G > A, p.Arg82Glu).

From 6 months of age, the patient was initially treated with levetiracetam (75 mg twice daily), with subsequent addition of pyridoxine (100 mg morning, 200 mg evening), folinic acid (5 mg/day) and carbidopa-levodopa (2.5 mg twice daily). The patient tolerated and adhered to the treatment; however, little symptomatic improvement was noted and she unfortunately died at 3 years of age. The cause of death was unknown because the patient passed away at her local hospital.

The patient was managed by multiple teams at KFSH-RC. The pediatric nutrition team was responsible for optimizing nutrition and caloric intake, and the patient received Infatrini formula. The patient also underwent a swallowing assessment owing to poor oral motor skills. Based on the results from this assessment, long-term alternative feeding was recommended to ensure the safety and adequacy of feeding. The pediatric surgical team was involved in G-tube insertion and performance of a Nissen fundoplication procedure to treat gastro-esophageal reflux disease.

### Patient case 2

This female patient was born to consanguineous parents and had a positive family history of AADC deficiency: she had one sister who was known to be a carrier and two other siblings with similar symptoms. Subsequent whole exome sequencing testing has confirmed that the two siblings with similar symptoms have AADC deficiency caused by pathogenic variants in the *DDC* gene and one brother is a carrier (Figure 2B).

The patient had experienced hypoxic encephalopathy since birth resulting in a cerebral palsy-like phenotype. The patient subsequently displayed multiple signs and symptoms consistent with AADC deficiency, including lack of head control, tremor, global developmental delay, disturbed sleep, drooling, excessive sweating, hyperthermia, constipation, and intractable epilepsy. She was initially diagnosed with developmental delay and seizure disorder but was referred to a specialist center at 15 years of age for seizure and neurological management. A genetic diagnosis was obtained at 18 years of age, with a homozygous variant in exon 3 identified (c.206C > T, p.Thr69Met). A brain MRI scan at this time was normal, but an EEG showed evidence of mild-to-moderate diffuse encephalopathy and intermittent high amplitude slow waves in the bifrontal area intermixed with fast activity progressing to generalized spike wave discharges, which may explain the intractable epilepsy symptoms. This patient did not receive CSF neurotransmitter analysis because her family declined a lumbar puncture procedure.

The patient commenced treatment at 12 years of age and at the time of referral to KFSH-RC was receiving levetiracetam (1 g twice daily), phenytoin (300 mg in the evening) and lacosamide (100 mg twice daily). As of March 2022, the patient is 21 years of age. She is being managed by the adult neurology department at KFSH-RC, reviewed yearly, and is receiving treatment with clonazepam (0.5 mg morning, 1 mg afternoon) and levetiracetam (1 g twice daily). The patient is reported to be tolerant and adherent to treatment. Initial treatment resulted in mild symptomatic improvements, with a slight reduction in the frequency and severity of seizures. However, these changes were temporary; no long-term improvement in her symptoms has been observed. The patient is also being managed by the dermatology department for scalp psoriasis.

### Patient case 3

This male patient was born to consanguineous parents. No family history of AADC deficiency was noted; however, the patient's parents had previously experienced two intrauterine fetal deaths and a cousin has a developmental delay. Subsequently, a sibling was born and diagnosed with AADC deficiency within the first few months of life owing to the known family history (Figure 2C).

The patient first experienced symptoms of AADC deficiency at 6 weeks of age, including hypotonia, oculogyric crises, global developmental delay, hypoglycemia, and global tonic-clonic seizures. Other signs and symptoms subsequently noted include an abnormal startle reflex, insomnia, phlegm accumulation leading to several episodes of pneumonia, silent aspiration on thin and nectar-thickened liquids (no aspiration with honey-thickened liquids), excessive sweating, hyperthermia, gastro-esophageal reflux disease, and suspected epilepsy.

The patient was referred to KFSH-RC at 3 months of age for further investigation of hypotonia and hypoglycemia. He was initially reviewed by the pediatric endocrine team and subsequently the neurology, genetics, pediatric surgery, nutrition, and speech and language pathology departments have been involved in his care. G-tube feeding is currently his main source of nutrition.

The patient underwent two brain MRI scans. In the first, no definite intracranial abnormality was observed. In the second, motion-degraded, diffusion-weighted images suggested tiny non-specific foci of signal abnormality, which may represent minor markers of metabolic derangement. An EEG revealed a non-specific bioccipital abnormality often associated with sedative medication, or, rarely, cortical dysplasia. No epileptiform abnormality was observed. Typically, patients with AADC deficiency display normal activity in MRI or EEG examinations. The signalling abnormalities detected may be the cause of the global tonic-clonic seizures. Genetic testing was requested when the patient was 7 months of age and outsourced to the USA. Results were received ∼9 months later and identified a homozygous variant in exon 2 in the *DDC* gene (c.175G > A, p.Asp59Asn). Both parents were subsequently found to be heterozygous carriers for the identified variant. The patient also underwent a lumbar puncture at 18 months of age and a CSF neurotransmitter profile characteristic of AADC deficiency was observed, including low levels of 5-HIAA (7 nmol/L) and HVA (20 nmol/L), and elevated 3-OMD (1,171 nmol/L). A plasma AADC enzyme activity assay was requested; however, an incorrect sample type was sent to the laboratory and therefore no results were obtained.

Treatment with levetiracetam (50 mg/kg/day) was initiated when the patient was 7 months old and subsequently carbamazepine (15 mg/kg/day) was added. At ∼2 years of age, the patient was taken to the USA for a further opinion at a specialist center and his medication was switched to ethacridine lactate, pramipexole, baclofen, and diazepam. His seizures improved with pramipexole, although side effects (abnormal movements) were reported when the dose was increased. As of March 2022, the patient is currently 5 years old and is being treated with pyridoxine (100 mg/day) and pramipexole (0.125 mg three times daily) under the care of KFSH-RC and reviewed 1–2 times per year.

## Discussion

In this report, we describe three Saudi Arabian patients diagnosed with AADC deficiency and managed at KFSH-RC. All three patients presented shortly after birth with symptoms that substantially affected their development and quality of life. All had a homozygous variant in the *DDC* gene, and, where data were available, biochemical analyses supported the diagnosis of AADC deficiency. All patients had a gene variant leading to a single amino acid substitution, both patients 1 and 2 had variants in exon 3 of the *DDC* gene (Arg82Glu and Thr69Met, respectively) that codes for loop 1 of the protein that likely plays a role in the conversion from the apo to the holo form of the AADC enzyme (18, 19). Patient 3 had a variant in exon 2 (c.175G > A, p.Asp59Asn) that codes for the N-terminal domain, which despite not affecting residues at the enzyme active sites or in the key loop regions, has been previously reported as a pathogenic variant (16).

In all three cases, the patient was initially managed at a local hospital before referral to KFSH-RC. Two patients were referred in the first year of life, but the third (patient case 2) did not receive a specialist review until she was 15 years of age. Diagnostic delays were common to all three cases for several reasons, including delayed referral to a specialist center and considerable delays in receiving results from outsourced genetic tests. Delays were further exacerbated by the requirement to outsource CSF analysis. The KFSH-RC plans to introduce on-site CSF analysis to help minimize diagnostic delays. For one patient, the family declined lumbar puncture, which meant that the clinical team were dependent on the delayed genetic testing results rather than gaining an indication of the diagnosis from CSF metabolite testing. A similar diagnostic delay was reported in a case series of 23 patients from mainland China diagnosed with AADC deficiency. In this study, the average age of diagnosis was 13 months, despite patients largely displaying symptoms from the early months of life (19). In a larger international case series of patients with AADC deficiency (*n* = 63) published in 2020, the time from symptom onset to diagnosis decreased over the past decade, with patients >10 years of age experiencing a diagnostic delay of 8 months to 31 years, whereas most patients ≤10 years of age experienced a diagnostic delay of approximately 1 year (3). This is consistent with the cases reported here, with the two younger patients diagnosed in the second year of life and the older patient only obtaining a diagnosis at 18 years of age.

In order to improve early diagnosis of AADC deficiency, it is vital to raise awareness among primary care physicians that if a patient is presenting with hypotonia, oculogyric crises and developmental delay, the three most commonly reported initial symptoms of AADC deficiency (3), in the absence of abnormalities found using brain MRI or EEG, they should immediately be referred to a specialist at a tertiary care center for further diagnostic testing. Introducing a screening program to all primary care centers, such as the use of dried blood spot sampling to measure levels of 3-O-methyldopa, could also aid in reducing diagnostic delays (20). The European Commission has recently granted marketing authorization for PTC Therapeutics’ product Upstaza™ (eladocagene exuparvovec), an *in vivo* gene therapy for treating AADC deficiency through delivery of the *DDC* gene to the putamen. It is approved for patients aged 18 months of age and older (21). Achieving an early diagnosis will be particularly crucial once gene therapy becomes available to ensure prompt treatment of patients.

Medical treatment was largely focused on seizure control, with patient 1 and 3 also receiving targeted therapies (dopamine replacement therapy and pyridoxine). Epileptic seizures as a result of AADC deficiency are rare, to date, few case reports have reported seizures as a symptom (22). When seizures have been reported, they have been described as “generalised seizures” (23), “generalized tonic-clonic seizures” (24) or “myoclonic epilepsy” (25). Epileptic EEG activity has only been reported in around half the cases (11). Although all three patients were reported to tolerate and be adherent to their treatment regimens, no or limited symptomatic improvements were noted, with patient 1 passing away at only 3 years of age. This finding is consistent with larger international case series, which reported a significant childhood mortality risk for patients with AADC deficiency when the disease prevented attainment of development milestones (3). Patients 2 and 3 continued to experience considerable developmental delay and poor quality of life at the time of last follow-up.

In all three cases, patients received multidisciplinary care and their families were all offered genetic counseling in accordance with the guidelines (9). It is crucial for immediate family to receive genetic counseling: if both parents are carriers for the gene variant, there is a 1 in 4 risk of disease recurrence. At the KFSH-RC, genetic counseling is also offered to the extended family as a means to detect other potential AADC deficiency cases. For these three cases, management was complicated owing to the large distances that the patients lived from the tertiary center: patients 1, 2 and 3 were required to travel 800–1,000, 867 and 460 km, respectively. Owing to the rural location in which these patients lived, it was necessary for all three to travel by airplane to the KFSH-RC for follow-up appointments, with travel and stay funded by the Saudi Arabian government. This resulted in the patients only being reviewed infrequently. A similar challenge with patient visits has recently been reported in the Middle East for patients diagnosed with Duchenne muscular dystrophy, another rare genetic disease (26). There is an unmet need to improve outreach and communication between tertiary and primary care centers so that the patients’ local care team is educated on the disease and can help ensure that the patient and their family understand the importance of follow-up care and attendance at hospital appointments.

A strength of this study is that it highlights the diagnostic challenges faced by healthcare professionals in the Kingdom of Saudi Arabia who manage patients with this rare condition. Limitations are that it is based solely on patient cases and, as for all case reports, it is vulnerable to selection and recall bias.

The findings from this small case series corroborate previous case reports indicating that globally, patients with AADC deficiency frequently have a delayed diagnosis and that long-term clinical outcomes are generally poor. In Middle Eastern countries, increased disease awareness among primary and pediatric physicians, reduced time to obtain genetic and biochemical diagnostic test results, and an increase in the number of local specialists will be required to improve outcomes for patients with AADC deficiency.

## Data Availability

The datasets for this article are not publicly available due to concerns regarding participant/patient anonymity. Requests to access the datasets should be directed to the corresponding author.

## References

[1] Hyland K Reott M. Prevalence of aromatic L-amino acid decarboxylase deficiency in at-risk populations. Pediatr Neurol. (2020) 106:38–42. 10.1016/j.pediatrneurol.2019.11.022 32111562

[2] Hyland K Clayton PT. Aromatic amino acid decarboxylase deficiency in twins. J Inherit Metab Dis. (1990) 13(3):301–4. 10.1007/BF01799380 1700191

[3] Pearson TS Gilbert L Opladen T Garcia-Cazorla A Mastrangelo M Leuzzi V Aadc deficiency from infancy to adulthood: symptoms and developmental outcome in an international cohort of 63 patients. J Inherit Metab Dis. (2020) 43(5):1121–30. 10.1002/jimd.12247 32369189 PMC7540529

[4] Chien YH Chen PW Lee NC Hsieh WS Chiu PC Hwu WL 3-O-Methyldopa levels in newborns: result of newborn screening for aromatic L-amino-acid decarboxylase deficiency. Mol Genet Metab. (2016) 118(4):259–63. 10.1016/j.ymgme.2016.05.011 27216367

[5] Himmelreich N Montioli R Bertoldi M Carducci C Leuzzi V Gemperle C Corrigendum to “aromatic amino acid decarboxylase deficiency: molecular and metabolic basis and therapeutic outlook” [mol genet metab. 2019 may;127(1):12-22]. Mol Genet Metab. (2021) 134(1–2):216. 10.1016/j.ymgme.2021.06.010 34244047

[6] Fusco C Leuzzi V Striano P Battini R Burlina A, Delphi panel experts’ group Spagnoli C. Aromatic L-amino acid decarboxylase (AADC) deficiency: results from an Italian modified delphi consensus. Ital J Pediatr. (2021) 47(1):13. 10.1186/s13052-021-00954-4 33478565 PMC7819234

[7] Whitehead N Schu M Erickson S Croxford J Peters M Hyland K. Estimated prevalence of aromatic L-amino acid decarboxylase (AADC) deficiency in the United States, European union and Japan (poster). Annual congress of the European society for gene and cell therapy. Lausanne, Switzerland (2018).

[8] Hyland K Reott M. Estimated prevalence of aromatic L-amino acid decarboxylase (aadc) deficiency in at-risk population (poster). Annual meeting of the American society of gene and cell therapy. Chicago, IL (2018).

[9] Wassenberg T Molero-Luis M Jeltsch K Hoffmann GF Assmann B Blau N Consensus guideline for the diagnosis and treatment of aromatic L-amino acid decarboxylase (aadc) deficiency. Orphanet J Rare Dis. (2017) 12(1):12. 10.1186/s13023-016-0522-z 28100251 PMC5241937

[10] Pearson TS Gupta N San Sebastian W Imamura-Ching J Viehoever A Grijalvo-Perez A Gene therapy for aromatic L-amino acid decarboxylase deficiency by MR-guided direct delivery of Aav2-aadc to midbrain dopaminergic neurons. Nat Commun. (2021) 12(1):4251. 10.1038/s41467-021-24524-8 34253733 PMC8275582

[11] Pons R Ford B Chiriboga CA Clayton PT Hinton V Hyland K Aromatic L-amino acid decarboxylase deficiency: clinical features, treatment, and prognosis. Neurology. (2004) 62(7):1058–65. 10.1212/wnl.62.7.1058 15079002

[12] Brun L Ngu LH Keng WT Ch'ng GS Choy YS Hwu WL Clinical and biochemical features of aromatic L-amino acid decarboxylase deficiency. Neurology. (2010) 75(1):64–71. 10.1212/WNL.0b013e3181e620ae 20505134

[13] Graziano C Wischmeijer A Pippucci T Fusco C Diquigiovanni C Noukas M Syndromic intellectual disability: a new phenotype caused by an aromatic amino acid decarboxylase gene (DDC) variant. Gene. (2015) 559(2):144–8. 10.1016/j.gene.2015.01.026 25597765

[14] Opladen T Cortes-Saladelafont E Mastrangelo M Horvath G Pons R Lopez-Laso E The international working group on neurotransmitter related disorders (Intd): a worldwide research project focused on primary and secondary neurotransmitter disorders. Mol Genet Metab Rep. (2016) 9:61–6. 10.1016/j.ymgmr.2016.09.006 27830117 PMC5094101

[15] Kojima K Nakajima T Taga N Miyauchi A Kato M Matsumoto A Gene therapy improves motor and mental function of aromatic L-amino acid decarboxylase deficiency. Brain. (2019) 142(2):322–33. 10.1093/brain/awy331 30689738 PMC6377184

[16] Tseng CH Chien YH Lee NC Hsu YC Peng SF Tseng WI Gene therapy improves brain white matter in aromatic L-amino acid decarboxylase deficiency. Ann Neurol. (2019) 85(5):644–52. 10.1002/ana.25467 30864153

[17] Tai CH Lee NC Chien YH Byrne BJ Muramatsu SI Tseng SH Long-Term efficacy and safety of eladocagene exuparvovec in patients with aadc deficiency. Mol Ther. (2022) 30(2):509–18. 10.1016/j.ymthe.2021.11.005 34763085 PMC8822132

[18] Montioli R Dindo M Giorgetti A Piccoli S Cellini B Voltattorni CB. A comprehensive picture of the mutations associated with aromatic amino acid decarboxylase deficiency: from molecular mechanisms to therapy implications. Hum Mol Genet. (2014) 23(20):5429–40. 10.1093/hmg/ddu266 24865461

[19] Wen Y Wang J Zhang Q Chen Y Bao X. The genetic and clinical characteristics of aromatic L-amino acid decarboxylase deficiency in mainland China. J Hum Genet. (2020) 65(9):759–69. 10.1038/s10038-020-0770-6 32409695 PMC7387242

[20] Brennenstuhl H Kohlmuller D Gramer G Garbade SF Syrbe S Feyh P High throughput newborn screening for aromatic L-amino-acid decarboxylase deficiency by analysis of concentrations of 3-O-methyldopa from dried blood spots. J Inherit Metab Dis. (2020) 43(3):602–10. 10.1002/jimd.12208 31849064

[21] PTC Therapeutics. UpstazaTM (2022). Available at: https://ir.ptcbio.com/news-releases/news-release-details/upstazatm-granted-marketing-authorization-european-commission (Accessed September 23, 2022).

[22] Ito S Nakayama T Ide S Ito Y Oguni H Goto Y Aromatic L-amino acid decarboxylase deficiency associated with epilepsy mimicking non-epileptic involuntary movements. Dev Med Child Neurol. (2008) 50(11):876–8. 10.1111/j.1469-8749.2008.03094.x 18754761

[23] Swoboda KJ Saul JP McKenna CE Speller NB Hyland K. Aromatic L-amino acid decarboxylase deficiency: overview of clinical features and outcomes. Ann Neurol. (2003) 54(Suppl 6):S49–55. 10.1002/ana.10631 12891654

[24] Anselm IA Darras BT. Catecholamine toxicity in aromatic L-amino acid decarboxylase deficiency. Pediatr Neurol. (2006) 35(2):142–4. 10.1016/j.pediatrneurol.2006.01.008 16876014

[25] Hsieh HJ Lin SH Liu HM. Visualisation of impaired dopamine biosynthesis in a case of aromatic L-amino acid decarboxylase deficiency by co-registered 18f-Fdopa pet and magnetic resonance imaging. Eur J Nucl Med Mol Imaging. (2005) 32(4):517. 10.1007/s00259-004-1618-6 15821966

[26] Alghamdi F Al-Tawari A Alrohaif H Alshuaibi W Mansour H Aartsma-Rus A Case report: the genetic diagnosis of duchenne muscular dystrophy in the Middle East. Front Pediatr. (2021) 9:716424. 10.3389/fped.2021.716424 34595143 PMC8476401

